# Effect of step frequency on leg stiffness during running in unilateral transfemoral amputees

**DOI:** 10.1038/s41598-020-62964-2

**Published:** 2020-04-06

**Authors:** Hiroaki Hobara, Hiroyuki Sakata, Yuta Namiki, Genki Hisano, Satoru Hashizume, Fumio Usui

**Affiliations:** 1 National Institute of Advanced Industrial Science and Technology, Tokyo, Japan; 2 Tokyo University of Science, Chiba, Japan; 3 Tokyo Institute of Technology, Tokyo, Japan; 4 Tetsudou Kousaikai Foundation, Tokyo, Japan

**Keywords:** Biomedical engineering, Neurophysiology, Disability

## Abstract

Spring-like leg behavior is a general feature of mammalian bouncing gaits, such as running and hopping. Although increases in step frequency at a given running speed are known to increase the stiffness of the leg spring (*k*_leg_) in non-amputees, little is known about stiffness regulation in unilateral transfemoral amputees. In this study, we investigated stiffness regulation at different step frequencies at a given running speed in unilateral transfemoral amputees. We recruited nine unilateral transfemoral amputees wearing running-specific prostheses. They were asked to perform the action of running across a range of step frequencies (±20, ±15, ±10, ±5, and 0% of their preferred step frequency) at a given speed on an instrumented treadmill. The *k*_leg_ values were calculated using ground reaction force data in both the affected and unaffected limbs. It was found that *k*_leg_ increased with increasing step frequency for the unaffected limb, but not for the affected limb. Consequently, the unilateral transfemoral amputees attained the desired step frequency in the unaffected limb, but were unable to match the three highest step frequencies using their affected limbs. These results suggest that the stiffness regulation strategy during running differs between the affected and unaffected limbs.

## Introduction

**Figure 1:**
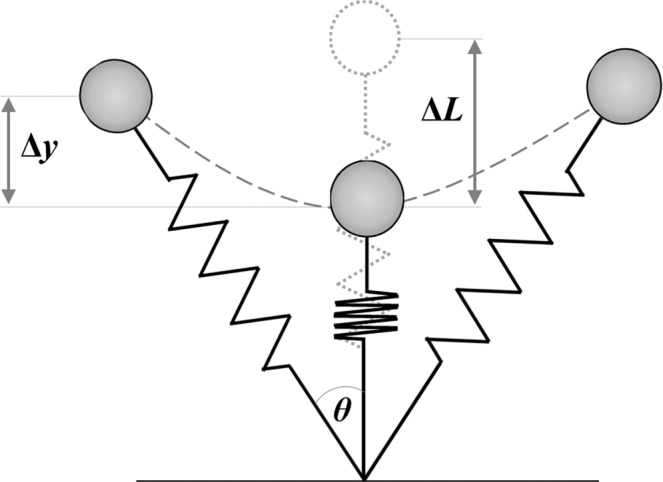
Spring–mass model for running. The leg spring is compressed during the first half of the stance phase and rebounds during the second half. The maximal vertical displacement of the center of mass and the leg spring compression during ground contact are represented by Δ*y* and Δ*L*, respectively. Half of the angle swept by the leg spring during the ground contact is denoted by *θ*.

The spring–mass model is widely used to quantify spring-like leg functions in mammalian bouncing gaits, such as hopping and running (Fig. 1)^1–3^. The model consists of the subject’s body mass and a massless linear leg spring. In the model, the stiffness of the leg spring (leg stiffness; *k*_leg_) has been shown to change depending on the demand. For example, *k*_leg_ is invariant over a wide range of running speeds^2^, but is increased with step frequency at a given running speed^4^. As reported in previous studies, humans increase *k*_leg_ to accommodate increases in step frequencies (*f*_step_) during running at a given speed^4–6^. Further, humans offset the increased *k*_leg_ in the mechanical behavior of the spring–mass system by decreasing the angle swept by the leg spring (*θ*) at a higher *f*_step_^4^. As a result, the vertical stiffness of the spring–mass system (*k*_vert_) increases, the vertical displacement of the center of mass (COM) during the ground contact time (*t*_c_) decreases, and the system bounces off the ground in less time as *f*_step_ is increased^4^. Because spring-like leg behavior is a general feature of mammalians bouncing gaits, an improved understanding of the frequency-dependent modulation of leg stiffness regulation will provide insight into the neuromechanical principles of legged locomotion in humans.

The use of carbon-fiber running-specific prostheses (RSPs) have allowed lower-extremity amputees to regain running ability by providing spring-like leg function in the affected limb. Stiffness regulation during running across a range of *f*_step_ has been examined in unilateral transtibial amputees, where *k*_leg_ was increased in the unaffected limb but was unchanged in the affected limb^7^. However, little is known about stiffness regulation over a wide range of *f*_step_ during running in unilateral transfemoral amputees (TFAs). Because *f*_step_ is associated with running velocity, physiological responses, and potential injury risks^8–10^, knowledge of how unilateral TFAs adjust stance leg mechanics for increasing *f*_step_ at a given running velocity may help in developing effective running-gait rehabilitation and individualized specifications of RSPs. Therefore, this study investigated stiffness regulation during running at different *f*_step_ values at a given running speed in unilateral TFAs wearing RSPs.

A recent finding showed that an athlete with unilateral transtibial amputation was unable to match relatively lower and higher hopping frequencies as a consequence of the invariant leg spring stiffness of the affected limb during one-legged hopping^11^. Further, in unilateral transtibial amputees, Oudenhoven *et al*. demonstrated that *k*_leg_ of the affected limb did not change across a range of *f*_step_ at a given running speed^7^. Because both the biological knee and ankle joints are missing in the affected limbs, the asymmetric adjustment of spring-like leg behavior is likely to be retained in unilateral TFAs. Thus, it was hypothesized that *k*_leg_ of unilateral TFAs would differ between the affected and unaffected limbs over a wide *f*_step_ range at a given running speed.

## Results

### Vertical ground reaction force–center-of-mass displacement curves

**Figure 2:**
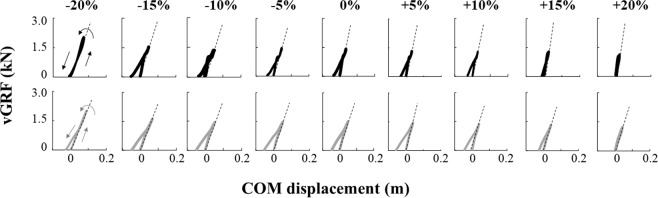
Time-normalized vGRF–COM displacement curves during ground contact while running at −20% to +20% of the preferred step frequency (0%). Black and gray curves indicate the unaffected and affected limb, respectively, recorded for one subject. The leg is compressed from the landing, and the vGRF is increased with COM displacement. The vGRF peaks at the midstance, and subsequently, the GRF decreases with the extension of the leg until take-off. The direction of the force–COM displacement curves is counter-clockwise in all conditions. The slopes (dotted lines) of these curves represent the vertical stiffness (*k*_vert_). *k*_vert_ is the slope of the vGRF–COM displacement curve in the leg compression phase.

Figure 2 depicts a typical example of the relationship between the vertical ground reaction force (vGRF) and center-of-mass (COM) displacement curves (recorded from one subject) during running in the range of −20% to +20% of the subject’s preferred *f*_step_. Both legs are compressed at touchdown, and vGRF is increased with COM displacement. The vGRF peaks at midstance, and subsequently, the vGRF decreases with the extension of the leg until take-off.

### Actual step frequency

**Figure 3:**
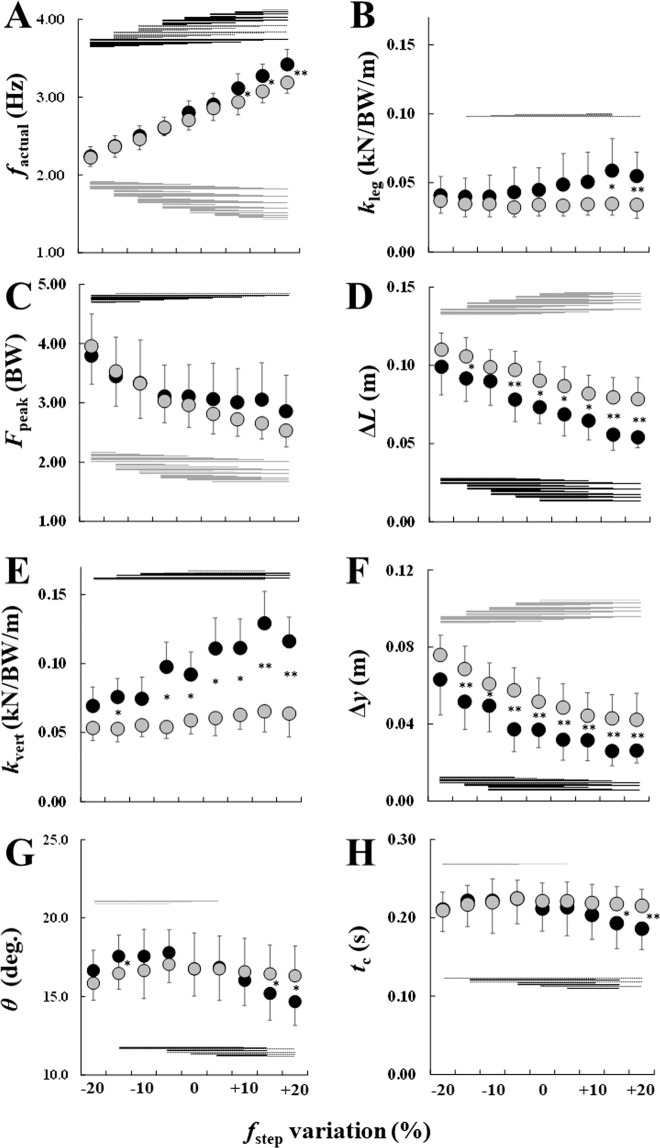
Comparisons of (**A**) *f*_actual_, (**B**) *k*_leg_, (**C**) *F*_peak_, (**D**) Δ*L*, (**E**) *k*_vert_, (**F**) Δ*y*, (**G**) *θ*, and (**H**) *t*_c_ across a range of step frequencies (*f*_step_). Black (unaffected leg) and gray (affected leg) circles are the means of the nine subjects. Asterisks (*, **) indicate significant differences between the unaffected and affected legs at *p* < 0.05 and 0.01, respectively. Black (unaffected leg) and gray (affected leg) horizontal lines indicate significant differences at *P* < 0.05 (dotted lines) and 0.01 (solid lines), respectively.

First, we determined the actual step frequency (*f*_actual_), which is defined as the inverse of the time from touchdown to contralateral touchdown. On average, the unaffected limb could match the metronome frequency within 3% at all designated values of *f*_step_. The affected limb could match the designated metronome frequency from −20% to +5% within the 3% criteria, but not at the three highest frequencies (+10%, +15%, and +20%), at which the actual step frequency (*f*_actual_) of the affected limb was lower than the designated frequency. We found a significant main effect of *f*_step_ on *f*_actual_, but no significant main effect of limb (Table 1). We also observed a significant interaction effect of *f*_step_ and limb on *f*_actual_ (Table 1). Simple main effects demonstrated that *f*_actual_ was significantly increased as *f*_step_ increased in both the affected and unaffected limbs; however, *f*_actual_ of the unaffected limb was significantly higher than the affected limb at +10%, +15%, and +20% *f*_step_ (Fig. 3A). Consequently, the differences in *f*_actual_ between the affected and unaffected limbs was greater for higher *f*_step_ (especially at +10%, +15%, and +20%) than for lower *f*_step_ (Fig. 3A).

**Table 1:** Results of the two-way repeated measures ANOVA. *F* values and corresponding *P* values are presented for all spring-mass parameters.

	*f*_step_		Limb		*f*_step_ × Limb	
	*F*	Sig.	*F*	Sig.	*F*	Sig.
*f*_actual_ (Hz)	909.92	*P* = < 0.01	3.56	*P* = 0.10	11.4	*P* = < 0.01
*k*_leg_ (kN/BW/m)	8.83	*P* = < 0.01	4.88	*P* = 0.06	8.87	*P* = < 0.01
*F*_peak_ (N/BW)	40.61	*P* = < 0.01	0.90	*P* = 0.37	14.28	*P* = < 0.01
Δ*L* (m)	96.09	*P* = < 0.01	9.14	*P* = < 0.05	5.06	*P* = < 0.01
*k*_vert_ (kN/BW/m)	20.04	*P* = < 0.01	9.41	*P* = < 0.05	6.38	*P* = < 0.05
Δ*y* (m)	62.38	*P* = < 0.01	20.05	*P* = < 0.01	0.94	*P* = 0.49
*θ* (deg.)	9.29	*P* = < 0.01	0.01	*P* = 0.96	29.91	*P* = < 0.01
*t*_c_ (s)	9.69	*P* = < 0.01	1.37	*P* = 0.28	20.4	*P* = < 0.01

### Leg stiffness, peak vGRF, and peak leg length compression

For all test subjects, the main effect of *f*_step_ on *k*_leg_ was significant, but no significant main effect for the limbs was found (Table 1). Statistical analysis revealed a significant interaction effect on *k*_leg_ (Table 1). As a result, the test of the simple main effect indicated an increase in *k*_leg_ from the lowest to highest *f*_step_ in the unaffected limb, but not in the affected limb. A significant difference in *k*_leg_ was noted between −15% and +20%, −10% and +15%, −5% and +15%, and +10% and +15% in the unaffected limb. Further, *k*_leg_ was significantly smaller in the affected limb than in the unaffected limb at +15% and +20% *f*_step_ (Fig. 3B). The peak vGRF (*F*_peak_) showed significant main effects of *f*_step_, but no significant main effect of the limb (Table 1). A significant interaction effect on *F*_peak_ was also observed (Table 1). Because of the simple main effect, *f*_step_ had a significant effect on *F*_peak_, whereas no significant limb effect was observed on *F*_peak_. However, the differences in *F*_peak_ between the affected and unaffected limbs was more noticeable for higher *f*_step_ (especially +10%, +15%, and +20%) than for lower *f*_step_ (Fig. 3C). There was a significant main effect of *f*_step_, limbs, and interaction effects on the peak leg spring compression (Δ*L*; Table 1 and Fig. 3D). A test of the simple main effects identified a decrease in Δ*L* over a wide *f*_step_ range for both limbs. Furthermore, we found that Δ*L* was significantly higher in the affected limb than in the unaffected limb at −15% and from −5% to +20% *f*_step_. Consequently, differences in Δ*L* between the affected and unaffected limbs were greater for relatively higher *f*_step_.

### Vertical stiffness and peak vertical COM displacement

Statistical analysis revealed significant main effects of *f*_step_ and the limb on vertical stiffness (*k*_vert_), as well as the interaction effect (Table 1). Tests of the simple main effects identified an increase in *k*_vert_ from the lowest to the highest *f*_step_ for the unaffected limb, but no changes in that for the affected limb (Fig. 3E). *k*_vert_ of the affected limb was significantly smaller than that of the unaffected limb at −15% and from −5% to +20% (Fig. 3E). There was a significant main effect of *f*_step_ and the limb on the peak vertical displacement of COM (Δ*y*; Table 1). However, no significant interaction effect between *f*_step_ and the limb on Δ*y* was found (Table 1). Statistical analysis revealed that Δ*y* was significantly decreased with increasing *f*_step_ in both limbs. It was also found that Δ*y* of the affected limb was significantly greater than that of the unaffected limb at all prescribed *f*_step_ except −20% (Fig. 3F).

### Half-angle swept by the leg spring and ground contact time

Significant main effects of *f*_step_ were noted on the half-angle swept by the leg spring (*θ*), but no main effect of limb was observed on *θ* (Table 1). A significant interaction effect on *θ* was also identified (Table 1). The post hoc analysis revealed that *θ* was slightly increased from −20% to +5% *f*_step_ in the affected limb only. On the other hand, *θ* was significantly decreased from lower to higher *f*_step_ in the unaffected limb (Fig. 3G). Furthermore, we observed that *θ* was significantly greater in the affected limb at +15% and +20% *f*_step_ (Fig. 3G). We also identified a significant main effect of *f*_step_ on contact time (*t*_c_; Table 1). Additionally, a significant interaction effect between *f*_step_ and the limb on *t*_c_ was noted. However, no significant main effect of the limb was observed. The simple main effects showed that *t*_c_ in the affected limb increased from −20% to +5% but remained nearly constant until +20% *f*_step_ (Fig. 3H). On the other hand, *t*_c_ in the unaffected limb remained nearly constant when the subject ran at a relatively lower *f*_step_ but decreased at a relatively higher *f*_step_. Compared with that of the unaffected limb, *t*_c_ of the affected limb was significantly longer at +15% and +20% *f*_step_ (Fig. 3H).

## Discussion

We found that the unaffected limb could match the metronome frequency within 3% at all designated step frequencies. Further, we found that *k*_leg_ and *k*_vert_ of the unaffected limb were increased with higher *f*_step_ (Fig. 3B-E). These results are consistent with past findings suggesting that *k*_leg_ and *k*_vert_ are increased with higher *f*_step_ during running in non-amputees^4–6^ and in the unaffected limbs of unilateral transtibial amputees^7^. However, unilateral TFAs could not match the three fastest values of *f*_step_ (+10%, +15%, and +20% *f*_step_) with their affected limbs, and the affected limb maintained nearly constant *k*_leg_ and *k*_vert_ values over a wide *f*_step_ range of. The results of this study are also consistent with those of a previous study, which showed that *k*_leg_ of affected limbs in unilateral transtibial amputees remained virtually constant across a range of *f*_step_ at a given running speed^7^. Therefore, the results support our hypothesis that the *k*_leg_ of unilateral TFAs would differ between the affected and unaffected limbs across a range of *f*_step_ at a given running speed.

In this study, increases in *k*_leg_ and *k*_vert_ of the unaffected limb at higher *f*_step_ were accompanied by decreases in Δ*L*, Δ*y*, *θ*, and *t*_c_ (Fig. 3D,F,G,H). The current results agree with a past finding that, as the stiffness of the spring–mass system increases, the vertical displacement of the COM during the ground contact phase decreases, and the system facilitates bouncing off the ground in a shorter time at higher frequencies^4^. On the other hand, we found invariant *k*_leg_ and *k*_vert_ of the affected limb across a range of *f*_step_ (Fig. 3B–E). A possible explanation for the invariant *k*_leg_ and *k*_vert_ of the affected limb with increasing *f*_step_ is the inability to regulate the affected limb’s stiffness. A past finding demonstrated that *k*_leg_ of the affected limb in athletes with unilateral transtibial amputation was constant with increasing *f*_step_ at a given running speed^7^. According to this study, athletes with unilateral transtibial amputation would make the affected limb as stiff as possible to conform to the natural frequency of the RSP to the extent possible. Further, a recent finding demonstrated that an athlete with unilateral transtibial amputation could not follow relatively higher and lower hopping frequencies as a consequence of the invariant *k*_vert_ of the affected limb^11^. As suggested by a previous study^12^, the stiffness of the RSP including the prosthetic knee likely dictates the whole-leg stiffness during running, leading to invariant *k*_leg_ and *k*_vert_ of the affected limb across a range of *f*_step_ at a given speed.

Surprisingly, the subjects in our study could match the targeted *f*_step_ from a relatively lower *f*_step_ to the preferred *f*_step_ (0%) without changing *k*_leg_ and *k*_vert_ (Fig. 3B–E). To do so, the subjects decreased *θ* and *t*_c_ in these conditions (Fig. 3G,H). However, the compensatory strategy of modifying *θ* and *t*_c_ in the affected limb might be insufficient at relatively higher *f*_step_ because of the mechanical compression–decompression properties of the RSP^13^. Further, we also observed that *F*_peak_ did not change from the preferred *f*_step_ to a higher *f*_step_ (Fig. 3C). This may be because unilateral TFAs must maintain the minimum vGRF and the corresponding impulse to rebound from the ground under the gravitational environment. These constraints limit the magnitude of the mechanical compression–decompression properties of the RSP; therefore, the affected limb would be unable to reduce *t*_c_ any further, especially at relatively higher *f*_step_. Consequently, unilateral TFAs would be unable to match the three highest *f*_step_ conditions (+10%, +15%, and +20% *f*_step_) with their affected limbs.

As shown in Fig. 3B–E, *k*_leg_ and *k*_vert_ were generally lower in the affected limb than in the unaffected limb. Recent studies also demonstrated that *k*_leg_ and *k*_vert_ of the affected limb were lower than those of the unaffected limb in unilateral TFAs wearing RSPs during running^14,15^. Further, the smaller values of *k*_leg_ and *k*_vert_ of the affected limb were mainly associated with greater Δ*L* and Δ*y*, compared to the unaffected limb, but not with *F*_peak_ (Fig. 3C,D,F). As shown in Eqs. (2) and (3), Δ*L* is a function of Δ*y*, *L*_0_, *θ*, and *t*_c_ when the foot is on the ground. Therefore, it is plausible that the higher Δ*L* in the affected limb than the unaffected limb may be attributed to (1) larger *θ* and longer *t*_c_^16^ (Fig. 3G,H), (2) 5% longer leg spring length (*L*_0_) of the affected limb (Table 2), (3) mechanical properties of RSPs^12^, (4) other residual structures, such as the socket–stump interface and/or larger pelvic obliquity of the frontal plane in the proximal hip joint, or any combination of these factors.

**Table 2:** Subject characteristics.

Subject	Sex	Age (years)	Height (m)	Total mass (kg)	Amputated limb	Time since amputation (years)	prosthetic knee	RSP model	Affected *L*_0_ (m)	Unaffected *L*_0_ (m)	Trial speed (m/s)	Preferred *f*_step_ (Hz)
1	F	20	1.56	56.5	Right	5.7	3S80	Runner 1E91 (cat.3)	0.85	0.84	2.39	2.88
2	F	21	1.49	46.7	Right	10.0	3S80	Sprinter 1E90 (cat.2)	0.85	0.76	1.94	3.00
3	F	20	1.62	45.2	Left	3.5	3S80	Sprinter 1E90 (cat.3)	0.85	0.84	2.14	2.68
4	M	17	1.77	86.0	Right	3.5	3S80	Sprinter 1E90 (cat.4)	0.93	0.89	2.75	2.93
5	M	23	1.68	56.3	Left	20.0	3S80	Sprinter 1E90 (cat.3)	0.89	0.84	2.31	2.73
6	M	34	1.61	58.7	Left	21.0	3S80	Runner 1E91 (cat.5)	0.85	0.82	2.25	2.85
7	M	27	1.75	70.4	Right	6.2	3S80	Runner 1E91 (cat.4)	0.96	0.89	2.86	2.70
8	M	36	1.61	59.8	Right	17.9	3S80	Runner 1E91 (cat.3)	0.87	0.81	2.11	2.53
9	M	31	1.65	59.7	Right	3.0	Cheetah Knee	Runner 1E91 (cat.2)	0.88	0.87	2.00	2.65
Mean		25.44	1.64	59.91		10.09			0.88	0.84	2.31	2.77
(SD)		(6.45)	(0.08)	(11.6)		(7.1)			(0.04)	(0.04)	(0.30)	(0.14)

A better understanding of spring-like leg behavior and stiffness regulation in this population will provide insight into the underlying biomechanics and control mechanism of leg stiffness in humans and would assist in developing design parameters for spring-based prostheses for running^4^. For example, a past finding demonstrated the asymmetric *k*_leg_ and *k*_vert_ modulation of unilateral TFAs for a range of running speeds^14^. In our study, asymmetric *k*_leg_ and *k*_vert_ modulation is observed across a range of *f*_step_ at a given speed (Figure B and E). Hence, the past finding and present study both suggest that spring-like leg behavior and stiffness regulation during running differ between the affected and unaffected limbs in unilateral TFAs. In other words, unilateral TFAs wearing RSPs would adopt limb-specific control strategies to accommodate the demands of specific activities. Because increased *k*_leg_ and *k*_vert_ during running and hopping may be related to bone-related injuries such as knee osteoarthritis and stress fractures^17^, coaches and practitioners should consider limb-specific injury risks and control mechanisms during running in unilateral TFAs for running-gait rehabilitation and training regimes^18^. Additionally, the observed asymmetric *k*_leg_ and *k*_vert_ modulation could be improved by changing the prosthetic mechanical properties and configurations. Indeed, previous studies suggested that RSP stiffness^12^ and prosthetic alignment^13^ could influence running performance through the regulation of stiffness in lower-extremity amputees. Although participants of the present study used their preferred RSP design and category of stiffness as well as prosthetic alignment, this may have induced the asymmetric *k*_leg_ and *k*_vert_ modulation. Therefore, the current results may contribute to providers’ and patients’ decision-making regarding the types and properties of the RSPs that they will employ for running.

There are certain considerations that must be acknowledged when interpreting the results of the current study. First, although we quantified the leg stiffness, which represents the overall stance leg mechanics, the natural frequency of the prosthetic foot and the stiffness of the prosthetic knee and foot were not determined in this study. As shown in Fig. 2, it seems that the use of a prosthetic knee and foot results in a large amount of energy loss (hysteresis) in the vGRF–COM displacement curves during ground contact. Therefore, as demonstrated by past findings^7,14,19^, the stiffness of each prosthetic component should be addressed to determine stiffness regulation during running using RSPs. Second, in our study, each participant ran at different *f*_step_ at a given running speed, which differed depending on the participant. This is because a one-speed trial is insufficient to obtain a wide *f*_step_ range for all participants. Instead, we used the normalized speed (40% of the estimated maximum speed); consequently, this normalized speed was sufficiently low for all participants to obtain a wide *f*_step_ range. However, stiffness regulation at different *f*_step_ in each participant might be affected by the trial speed. Thus, caution must be used in the interpretation and generalization of these findings.

## Conclusion

In summary, the results of this study suggest that (1) the affected limb of unilateral TFAs cannot modulate *k*_leg_ across a range of *f*_step_ at a given running speed, and (2) the *k*_leg_ regulation strategy differs between the affected and unaffected limbs. Between-limb asymmetry in the *k*_leg_ regulation strategy during running in unilateral TFAs may arise from their compensatory strategies and the mechanical constraints of their prosthesis properties.

## Methods

### Participants

In this study, we recruited nine TFAs who specialized in the 100-m sprint or long jump (Table 2). Five of the participants used the 1E91 Runner (categories 2 to 5, Ottobock, Duderstadt, Germany) and four participants used the 1E90 Sprinter (categories 2 to 4, Ottobock, Duderstadt, Germany) with rubber soles (Table 2). All of these participants belonged to track and field teams and had performed sprint training for more than five years. On average, their best recorded times in the 100-m sprint within the preceding year were 17.59 ± 2.15 s. Before the experiment, all participants (or guardians, in the case of participant 4) gave informed written consent approved by the local ethical committee. The study was ethically approved by the Institutional Review Board of our institution (Environment and Safety Headquarters, Safety Management Division, National Institute of Advanced Industrial Science and Technology) and conducted in accordance with the guidelines set out in the Declaration of Helsinki (1983).

### Tasks and experimental procedures

In this study, participants ran on an instrumented treadmill (FTMH-1244WA, Tec Gihan, Kyoto, Japan). First, as a familiarization period for instrumented treadmill running, we instructed all subjects to perform running and walking more than 5 min before the experiment^20^. Next, each participant performed a single bout of 20-s runs to determine the preferred *f*_step_ for running at 40% of their maximum speed (Table 2), which was estimated by dividing 100 m by their personal best time in a 100-m sprint^14^. 40% of the estimated maximum speed was chosen as the running speed because this was sufficiently low for the participants to obtain a wide *f*_step_ range. We determined the preferred *f*_step_ using 14 consecutive steps in the middle of the trial. In the present study, the *f*_step_ was defined as the inverse of the time from touchdown to the contralateral touchdown. Because previous studies varied the *f*_step_ range from −30% to +30% at a given running speed^4–7^, we asked participants to run with a digital metronome beat at nine values of *f*_step_: the preferred (0%), four below (−5%, −10%, −15%, and −20%), and four above the preferred *f*_step_ (+5%, +10%, +15%, and +20%). After a sufficient practice period at each *f*_step_, participants were asked to perform a single running trial for 20 s at each targeted *f*_step_ (in random order), with rest periods of 1–3 min between trials to minimize the effects of fatigue. On average, the running speed in this study was 2.31 ± 0.30 m/s for 40% of the maximum speed, and the preferred step frequency was 2.77 ± 0.14 Hz (Table 2).

### Data collection and analysis

vGRF was collected by two force platforms embedded in the instrumented treadmill (sampling at 1,000 Hz). According to a previous study^21^, a fourth-order zero-lag low-pass Butterworth filter with a cut-off frequency at 25 Hz was used to filter the vGRFs. Further, a 40-N threshold for further vGRF analysis was used^22–25^. Using the vGRF, the values of *f*_actual_, *t*_c_, and *F*_peak_ in both the unaffected and affected limbs were determined. The representative value of stiffness was determined by averaging five consecutive steps that were within 3% of the target *f*_step_. The *k*_leg_ (N/m) was computed as the ratio of *F*_peak_ to Δ*L* at the midstance. Thus,

1 $${k}_{\mathrm{leg}}={F}_{\mathrm{peak}}/\triangle L$$

with

2 $$\Delta L=\Delta y+{L}_{0}(1-\,\cos \,\theta )$$

where Δ*y* was calculated by twice integrating the vertical acceleration of the COM with respect to time^26^. The initial leg spring length (*L*_0_) was defined as the distance from the greater trochanter to the ground in standing position. Half of the angle swept by the leg spring during the first half of stance phase (*θ*) was calculated as

3 $$\theta ={\sin }^{-1}(u{t}_{c}/2{L}_{0})$$

where *u* is the average forward velocity and *t*_c_ is the ground contact time for each step. Finally, we calculated *k*_vert_ using the following formula:

4 $${k}_{\mathrm{vert}}={F}_{\mathrm{peak}}/\Delta y.$$

We calculated *k*_leg_ as the ratio of *F*_peak_ and Δ*L* (the ratio of *F*_peak_ and Δ*y* for *k*_vert_) between ground contact (initial leg compression phase) and the instant of *F*_peak_^11,14,27,28^. Because body mass influences the stiffness^2^, both *k*_leg_ and *k*_vert_ were normalized to the subject’s body weight (BW), which included the prosthesis.

### Statistics

A two-way repeated-measure analysis of variance (ANOVA) with two factors, *f*_step_ (nine levels) and limbs (two levels), was performed to compare the spring–mass parameters (*f*_actual_, *k*_leg_, *F*_peak_, Δ*L*, *k*_vert_, Δ*y*, *θ*, and *t*_c_) between the unaffected and affected limbs across a range of *f*_step_. Mauchly’s Test of Sphericity was used to ensure that the variances of the differences were comparable. If this assumption was violated, the Greenhouse–Geisser correction was applied. If a significant main effect was observed, a Bonferroni post hoc multiple comparison was performed. When the ANOVA indicated a significant interaction between the main effects, a test of the simple main effect was conducted. All statistical tests were performed at the 5% significance level using SPSS (IBM SPSS Statistics Version 19, SPSS Inc., Chicago, IL).

## Supplementary information

Supplementary information.

## Data Availability

The datasets generated and/or analyzed during the current study are available from the corresponding author on reasonable request.
